# GSK3 as a Master Regulator of Cellular Processes

**DOI:** 10.3390/ijms242115503

**Published:** 2023-10-24

**Authors:** Ralf Lichtinghagen, René Huber

**Affiliations:** Institute of Clinical Chemistry, Hannover Medical School, 30625 Hannover, Germany; lichtinghagen.ralf@mh-hannover.de

Since its initial purification and characterization as an enzyme negatively regulating glycogen synthase activity [1], and the identification of the highly similar paralogues α and β [2], glycogen synthase kinase (GSK) 3 has been proven to be a central modulator of manifold cellular processes targeting a plethora of molecules [3] and signaling pathways [4,5]. The central regulatory principle of GSK3 is the modulation of its activity by inhibitory phosphorylation in response to cellular stimulation with tumor necrosis factor, insulin, and multiple growth factors (amongst others) [6]. For Wnt (“wingless-type MMTV integration site family member”) glycoproteins, however, phosphorylation-independent mechanisms of GSK3 inactivation have been demonstrated based on GSK3 sequestration in vesicles [7] and the altered composition of GSK3-containing protein complexes [8]. Essential physiological functions directed by GSK3α and β comprise gene expression, metabolism, proliferation, differentiation/development, apoptosis, adhesion, and migration, amongst other things [6]. Accordingly, GSK3 is also involved in a variety of disorders, including neurological/neurodegenerative, metabolic, and inflammatory diseases, and different forms of cancer (also affecting angiogenesis and metastasis [4]) that are often associated with a dysregulation of GSK3 activity or enzyme–substrate interactions [9]. Therefore, GSK3 inhibition initially appeared to be a promising treatment strategy and several GSK3 inhibitors have been developed. In clinical trials, however, numerous approaches have yielded disappointing results, though some small molecule inhibitors are still under clinical investigation [6]. A major problem is the limited selectivity due to the high degree of homology between GSK3α and β, and paralog-selective inhibitors have been made available only recently [10]. In many biological contexts, however, GSK3 appears to mediate unexpected and sometimes even conflicting effects [6]. Thus, its specific behavior in a particular situation (e.g., during infections or cancer) is often difficult to predict and its role is still controversial. This Special Issue aims to enhance our understanding of the regulation of GSK3α and β under various conditions and the diverse ways in which both paralogues may modulate multiple physiological and pathophysiological processes within cells and organisms.

It is well established, for instance, that the activity of GSK3 proteins is predominantly controlled by inhibitory phosphorylation at Ser21 (GSK3α) and Ser9 (GSK3β). Alternative negative regulatory phosphorylation sites, however, such as Ser389, have also been described [11]. The relevance of this residue for the restriction of neuroinflammation—a topic deserving special attention in the context of mood and cognitive disorders [12]—is addressed in this Special Issue in the paper by Calvo and colleagues [13]. Based on an established Ser389 phosphorylation-deficient knock-in mouse model (Ser389Ala; [14]), the authors report that the inability to inactivate GSK3β via this mechanism led to an impaired activation of the nuclear factor κ-light-chain-enhancer of activated B cells (NF-κB-), but not the signal transducer and activator of transcription (STAT-) 3-associated signaling in the hippocampus. The affected mice were characterized by increased numbers of activated microglia, astrocytes, and infiltrated neutrophils in the brain, thus showing chronic basal neuroinflammation. Concomitantly, intraperitoneal lipopolysaccharide (LPS) injection—an approach widely used to induce neuroinflammation [15]—did not increase immune cell activation or infiltration, indicating an inadequate response towards pro-inflammatory stimulation. These effects appear to be brain-specific, since peripheral immune cells and the immune response towards LPS remained virtually unaffected, as reflected by normal cell counts and proper immune cell activation in the spleen [13].

The impact of endothelial GSK3α and β on atherosclerosis was addressed by two publications. Cai et al. highlighted that vascular calcification in atherosclerotic lesions (a process involving endothelial–mesenchymal transition [16]) can be attenuated through pharmacological inhibition or the endothelial-specific deletion of GSK3β. In the murine apolipoprotein E (Apoe) knock-out (KO) atherosclerosis model, pharmacological GSK3 inhibition significantly reduced aortic calcification, calcium load, and the induction of osteogenic as well as mesenchymal markers in atherosclerotic lesions [17]. Equivalent results were obtained in mice, showing the endothelium-specific KO of GSK3β in response to tamoxifen [17], and have also been observed in diabetic Ins2^Akita/+^ mice following GSK3β inhibition [18]. In the study by Mastrogiacomo et al., the impact of GSK3α and β KOs in endothelial cells (EC) and/or macrophages for atherogenesis was assessed. Therefore, atherosclerosis mouse models with an endothelial-specific KO of GSK3α or β should be used. The respective Tie2Cre strains, however, exhibited dual-specific GSK3 KO in EC and macrophages [19], thus matching earlier reports on Tie2Cre-directed gene deletion in both lineages [20]. While most of the parameters analyzed (such as plasma lipids and body weight) showed no GSK3-dependent differences, EC/macrophage-specific GSK3α (but not GSK3β) KO mice were characterized by significantly reduced plaque volume, EC activation, and monocyte/macrophage recruitment when compared to age-matched controls. To restrict the GSK3 KO to the endothelium, the authors performed bone marrow transplantation on the dual-specific KO mice, which restored GSK3α and β expression in the myeloid lineage. Subsequent analyses revealed that the EC-specific KO of GSK3α had comparable effects to the dual-specific KO. Again, GSK3β KO mice showed no such changes, together indicating that the significant pro-atherogenic effect observed was exclusively mediated by endothelial GSK3α [19], which fits well with the beneficial outcome of whole body [21] or myeloid-specific GSK3α KO [22] described before.

The next three studies presented in this Special Issue are dedicated to the impact of GSK3 modulation on central cellular processes, i.e., cell cycle progression [23], the management of amino acid (aa) deprivation [24], and metabolism [25]. Shenker et al., for instance, were interested in elucidating the cytotoxic properties of Aggregatibacter actinomycetemcomitans-derived cytolethal distending toxin (*Aa*Cdt) [23], a heterotrimeric bacterial toxin possessing both DNase and lipid phosphatase activity, which typically causes cell cycle arrest and apoptosis [26]. To focus on the molecular mechanisms of *Aa*Cdt-induced cell cycle arrest, this group used oral keratinocytes as a cell model due to their resistance towards Cdt-induced apoptosis and DNA damage response. In cell lines and primary human gingival keratinocytes, *Aa*Cdt significantly initiated a persistent, dose-dependent G2/M arrest in a phosphatase (but not DNase) activity-dependent manner (as shown before for lymphocytes and HeLa cells [27]), without an indication of DNA damage or elevated apoptosis. At the signaling level, this effect was associated with cyclin dependent kinase (CDK) 1 and Akt inhibition, as well as GSK3β activation. Correspondingly, *Aa*Cdt-induced G2/M arrest decreased in the presence of GSK3 inhibitors, indicating that the *Aa*Cdt-mediated inhibition of cell cycle progression involves GSK3β enzymatic activity [23]. In another paper, Loxha et al. had a closer look at GSK3α-regulated cancer cell survival under aa deprivation [24], a mechanism contributing to the resistance of cancer cells towards aa depletion (as practiced, for instance, by asparaginase application) by releasing protein-bound aa via enhanced GSK3α-driven protein degradation [28]. Hence, GSK3α knock-down (KD) in T-cell acute lymphoblastic leukemia and colorectal cancer cells massively reduced cell viability and protein ubiquitination in the presence of arginase, while cell death significantly increased [24]. In the latter, however, the pro-apoptotic aa deficiency-induced GCN2 (“general control nonderepressible 2”)–eukaryotic translation initiation factor (eIF) 2α–activating transcription factor (ATF) 4–C/EBP Homologous Protein (CHOP) axis [29] and cell cycle alterations were not involved. Subsequent RNA-sequencing analyses revealed that GSK3α KD cells surviving asparagine depletion were characterized by a downregulation of various ribosomal proteins (RP). Moreover, the suppression of specific RP in GSK3α KO cells was sufficient to protect them from asparaginase-induced cell death, collectively suggesting a detrimental influence of these RP on GSK3α-dependent cancer cell survival under these conditions [24]. In the next study, metabolic regulation was revisited as one of the most prominent functions of GSK3. Zhang et al. identified a GSK3 homolog (termed DcGSK3) closely related to GSK3 genes of the Hemptera species in *Diaphorina citri* Kuwayama (also known as Asian Citrus Psyllid) [25], a hemipteran bug acting as a vector for *Candidatus Liberibacter asiaticus*, the bacterium causing citrus greening disease [30]. DcGSK3 expression could be demonstrated among all tissues and developmental stages analyzed, with the highest levels in the head and during the egg phase, respectively. Following the microinjection of double stranded DcGSK3 RNA (dsDcGSK3) into fifth-instar *D. citri* nymphs to induce RNA interference, a significant reduction in DcGSK3 mRNA levels was accompanied by conspicuous adult phenotypes (abnormal molting, malformations, enhanced mortality). Moreover, sugar, fatty acid, and chitin metabolism were significantly affected, as represented by reduced levels of glucose, trehalose, glycogen, and fatty acids, as well as the dysregulated expression of various fatty acid and chitin metabolism-associated genes. A subsequent transcriptome analysis confirmed that among the genes differentially expressed in the dsDcGSK3-treated group, most candidates were involved in metabolic, transport, and structure-forming processes, according to gene ontology terms and the Kyoto Encyclopedia of Genes and Genomes pathways. Thus, DcGSK3 appears to be a crucial player in orchestrating central aspects of *D. citri* development and metabolism [25].

Finally, the ability of GSK3β to control behavioral characteristics was examined. The article by Cho et al. describes how the attenuation of amphetamine (AMPH-)-induced locomotion by a fragment (i.e., a peptide comprising aa 55-102) of the cocaine- and amphetamine-regulated transcript (CART; [31]) is mediated by GSK3β inhibition [32]. Following the intraperitoneal injection of AMPH, rats showed strongly enhanced locomotor activity. As reported earlier [31], this hyperlocomotion was significantly reduced when the rats received a bilateral microinjection of the CART peptide 55-102 into the nucleus accumbens (NAcc) before AMPH application. In NAcc core tissue from AMPH-treated rats, decreased levels of activating Akt-Thr308 and inhibitory GSK3β-Ser9 as well as increased levels of activating glutamate receptor 1 (GluA1-) Ser845 phosphorylation were observed, and the effects were completely abolished by CART peptide pretreatment. The next experiments confirmed the necessity to restrict GSK3β’s enzymatic function for inhibiting AMPH-induced motion, since the co-injection of both the CART and the S9 peptide (a small competitor for GSK3β-Ser9 phosphorylation that restricts GSK3β inhibition [33]) led to the abolishment of the CART-peptide-dependent inhibition of locomotor activity, a phenomenon associated with reduced GSK3β-Ser9 and increased GluA1-Ser845 phosphorylation. In consequence, the authors argue for a regulatory mechanism in which accumbal application of the CART peptide may prevent the cascade of AMPH-induced Akt inactivation, GSK3β activation, GluA1 phosphorylation, and subsequent locomotive hyperactivity [32].

Collectively, the seven studies published in this Special Issue shed new light on the multifaceted roles of GSK3 in cells, tissues, and organs and on the multiple levels of regulation to which the kinase is exposed, but also exerts, and the variety of (patho-) physiological conditions in which this bustling enzyme is involved. The present reports also substantiate the picture of GSK3 as a quite enigmatic molecular player that may throw the dice for beneficial as well as deleterious effects, making this kinase an equally tempting and difficult target for the clinical application of pharmacological interventions.
